# Recombinant Proteins-Based Strategies in Bone Tissue Engineering

**DOI:** 10.3390/biom12010003

**Published:** 2021-12-21

**Authors:** Marina Paulini, Iván Nadir Camal Ruggieri, Melina Ramallo, Matilde Alonso, José Carlos Rodriguez-Cabello, Pedro Esbrit, João Paulo Mardegan Issa, Sara Feldman

**Affiliations:** 1 Faculdade de Odontologia de Ribeirao Preto, University of Sao Paulo, Sao Paulo 05508-060, Brazil; marina.paulini@usp.br (M.P.); jpmissa@forp.usp.br (J.P.M.I.); 2 LABOATEM, Biology and Tissue Engineering Laboratory, School of Medicine, National Rosario University, Rosario 2011, Argentina; ivannadircamalruggieri@gmail.com (I.N.C.R.); mramallo96@gmail.com (M.R.); 3 BIOFORGE, Group for Advanced Materials and Nanobiotechnology, CIBER-BBN, University of Valladolid, 47011 Valladolid, Spain; malonso@bioforge.uva.es (M.A.); roca@bioforge.uva.es (J.C.R.-C.); 4 Area Osteoarticular, Instituto de Investigación Sanitaria (IIS)-Fundación Jiménez Díaz, 28040 Madrid, Spain; pesbrit@gmail.com

**Keywords:** recombinant proteins, bone, tissue engineering, BMP-2, scaffold

## Abstract

The increase in fracture rates and/or problems associated with missing bones due to accidents or various pathologies generates socio-health problems with a very high impact. Tissue engineering aims to offer some kind of strategy to promote the repair of damaged tissue or its restoration as close as possible to the original tissue. Among the alternatives proposed by this specialty, the development of scaffolds obtained from recombinant proteins is of special importance. Furthermore, science and technology have advanced to obtain recombinant chimera’s proteins. This review aims to offer a synthetic description of the latest and most outstanding advances made with these types of scaffolds, particularly emphasizing the main recombinant proteins that can be used to construct scaffolds in their own right, i.e., not only to impregnate them, but also to make scaffolds from their complex structure, with the purpose of being considered in bone regenerative medicine in the near future.

## 1. Introduction

Bone tissue constitutes approximately 18% of body weight and performs, among others, basic functions such as support, internal organs protection, and assistance in movement since skeletal muscles are attached to the bones and, when contracting, pull on the bones for movement [1]. It is a self-repairing tissue, capable of adapting its mass, shape, and properties to changing mechanical requirements and withstanding physical activity impacts [2,3,4].

Bone tissue has the following main types of cells [5,6]: (a) osteogenic cells, non-specialized stem cells derived from mesenchyme, the tissue from which all connective tissues originate; they are the only cells in this tissue that undergo cell division before transforming into osteoblasts, and can be found along the endosteum, the innermost portion of the periosteum, and in the intra-osseous ducts containing blood vessels; (b) osteoblasts, bone-forming cells that synthesize and secrete collagen fibers and other organic components necessary to build the osteoid matrix and initiate calcification; (c) osteocytes, which are produced when osteoblasts become trapped in the osteoid matrix, and respond to mechanical and hormonal stimuli, thereby coordinating the activities of osteoblasts and osteoclasts; and (d) osteoclasts, large cells produced by the union of several monocytes, with a high content of lysosome enzymes and acids that digest the components of the underlying cellular matrix, thus participating in processes of growth, maintenance and bone tissue repair [7,8,9,10,11].

The extracellular matrix is named the osteoid matrix and is made up of 25% water, 25% collagen fibers, and 50% crystallized mineral salts; one of the outstanding is hydroxyapatite, giving the bone tissue both strength and flexibility [12].

## 2. Bone Lesions and Regenerative Proposals

Orthopaedic surgery, odontostomatological surgery, neurosurgery, maxillofacial surgery, and others require the application of implant techniques in numerous situations, either because fracture healing does not occur in certain circumstances, or because critical injuries occur that do not fully regenerate [13]. The increase in fracture rates are related to changes in the lifestyles of the general population, as well as the shift of population pyramids towards older ages [14,15,16], causing a high impact socio-health problem.

Previous studies have shown encouraging results when using grafts of bone powder from cadaveric donors for bone fractures. However, it was concluded that although the risks of antigenic responses due to rejection associated with other types of implants were reduced, very controlled procurement and processing procedures are required, and there are often complications, which are rarely documented in the literature [17,18,19]. This has led researchers in these areas to delve into new regenerative tissue strategies, including those described below.

## 3. Tissue Engineering

Tissue engineering is a biotechnological area whose term emerged approximately 27 years ago, to define a multidisciplinary field of study encompassing material engineering and biomedical sciences. Therefore, it involves knowledge from various disciplines such as physics, chemistry, and biology, and seeks to reconstitute, replace and/or regenerate specific tissues or organs through the implementation of effective and practical materials, trying to maintain the existing residual structure, as well as to make tissue growth viable while acting as scaffolds that promote the growth of living tissues [20,21,22,23,24].

A variety of biodegradable materials have been developed, with great versatility to modify various parameters such as degradation rate, mechanical properties, and porosity, according to the characteristics required for each application. The scaffolds thus developed are called third generation scaffolds; they exhibit biological and physical properties compatible with the in vitro and in vivo physiological conditions of the damaged tissue. In other words, they are intended to provide a temporary support with sufficient mechanical integrity to maintain the structure, and then lead to proliferation, differentiation, and cellular biosynthesis specific to the site where it was implanted. To do so, it must exhibit properties such as being non-toxic, biocompatible, and biodegradable, promote and reorganize the desired cells, have adequate porosity for supporting cell seeding, and be interconnected. Third generation biomaterials to be considered as such must combine properties of biodegradation and bioactivity within the same material, the latter meaning that they must possess the capacity to induce, stimulate, provoke, or modulate a defined biological action in the host tissue. A bioactive material is one that enables a specific biological response at its interface with the surrounding tissues, by favoring their binding. These materials also seek to stimulate different cellular responses based on their surface characteristics, so that their function is therefore temporary, as the material is intended to be reabsorbed once the tissue function is restored. Tissue engineering applied to bone tissue repair must ensure that the materials used as scaffolds are potentially osteoinductive, i.e., capable of promoting the differentiation of progenitor cells into osteoblastic cells, and have osteointegrating properties, i.e., enabling integration with adjacent bone tissue, aiming to repair bone tissue with an intact biomechanical status [25].

An implant scaffold must present an architecture in which the cells are organized, promoting an initial biomechanical profile for tissue replacement until the cells produce an adequate extracellular matrix and the initial scaffold is degraded or metabolized to produce de novo tissue. The idea is to synthesize or implement a scaffold that is capable of supporting the initial loads and degrades gradually, transferring the loads progressively to the new bone. The degradation rate of a matrix used in bone tissue engineering should be slow, maintaining the mechanical resistance of the tissue itself until the bone tissue is regenerated and avoiding a second surgical intervention for implant removal. The scaffold materials must stimulate the appropriate cellular responses that will be gradually replaced by the new tissue [22,26].

Several research groups have developed scaffolds to be used as implants in bone lesions, some of them derived from natural products such as chitosan [27] and collagen [28,29,30], while other scaffolds have been produced by chemical synthesis [31,32]. Composite materials based on biodegradable polymers associated with other types of scaffolds have begun to be synthesized in the last decade, and they are of particular interest due to the fact that they offer an adequate balance between strength and toughness, beyond the properties of each of their individual components [33,34].

## 4. Recombinant Proteins in Bone Tissue Engineering

The advantage of using derivatives of natural biomolecules as scaffolds is that they could potentially be recognized by the cells surrounding the site of injury without generating any type of rejection, including their binding to specific cell surface receptors to generate immediate responses to start tissue formation by means of biochemical signals released from these cells [35].

The development of recombinant proteins as either scaffolds or scaffold components also offers the advantage of generating chimera proteins, i.e., the original human protein plus the sequence of some other protein that is considered useful for the subsequent process of tissue regeneration [36]. The following fundamental steps must take place in order to produce recombinant proteins: (a) natural DNA sequence determination; (b) recombinant DNA design; (c) vector cloning; (d) host organism engineering and transformation; (e) culturing/protein production; and (f) protein purification. So, the advantages of using recombinant proteins, and not obtained directly from nature as scaffolds or part of them, lie in the fact that: (a) recombinant proteins can be obtained homogeneously purified, according to state-of-the-art molecular biology strategies, avoiding contamination problems; (b) chimera proteins can be developed, adding specific recognition sequences; (c) they can be obtained on low-cost industrial scales according to the technologies developed so far; and (d) on certain occasions, sequences can be inserted that provide, in addition to the natural ones, certain characteristics that allow the recombinant to gelificate under certain circumstances, or to acquire certain structures that allow them to be used for direct application in regenerative processes, without the need to be attached to other types of scaffolds.

Given that some bone morphogenetic proteins (BMPs) are considered by many traumatologists to be the gold standard for application in bone regenerative medicine, we first briefly review them, as they are also present as part of other chimeric proteins mentioned later on [37].

Other ligand proteins, such as vascular endothelial growth factor (VEGF), platelet-derived growth factor (PDGF), insulin growth factor (IGF), etc., are biomolecules with a recognised role in bone tissue repair, and have been cloned and applied in tissue engineering experiments [38,39,40]. There is not much information in the literature that chimeric proteins containing sequences of these ligands in their structure have been synthesized. This work focuses on highlighting the main recombinant proteins that can be used to construct scaffolds in their own right, i.e., not only to impregnate them, but also to make scaffolds from the beginning.

## 5. Recombinant Human BMPs in Bone Tissue Engineering

**Figure 1:**
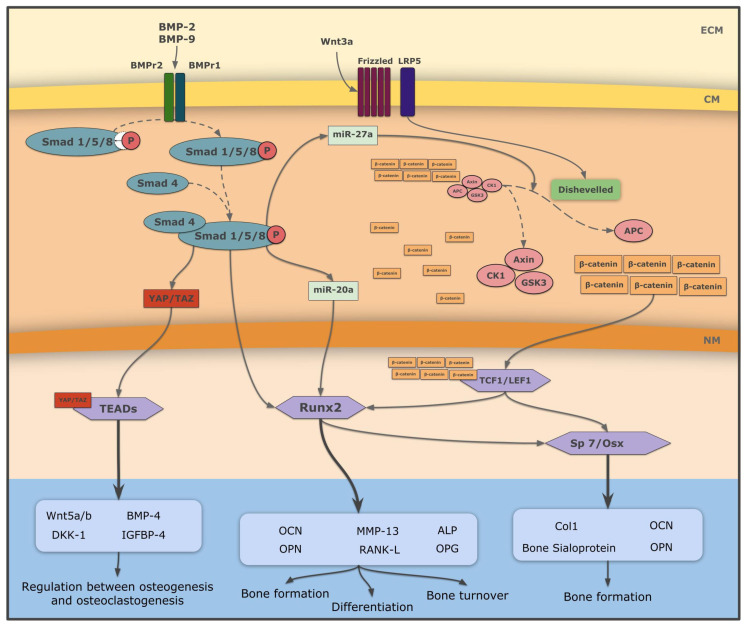
A brief summary of BMP-2 and BMP-9 signaling pathways, and their relation to the Wnt canonical pathway. *ECM*, extracellular matrix; *CM*, cell membrane; *NM*, nuclear membrane; *BMP-2*, bone morphogenetic protein 2; *BMP-4*, bone morphogenetic protein 4; *BMP-9*, bone morphogenetic protein 9; *BMPr2*, bone morphogenetic protein 2 receptor; *BMPr1*, bone morphogenetic protein 1 receptor; *miR*, microRNA; *miR-27a*, microRNA 27a; *miR20a*, microRNA 20a; *Wnt3A*, Wnt family member 3A; *Wnt5a/b*, Wnt family member 5a and 5b; *DKK-1*, Dickkopf-related protein 1; *LRP5*, low-density lipoprotein receptor-related protein 5; *YAP*, yes-associated protein; *TAZ*, transcriptional co-activator with PDZ-binding motif; *APC*, activated protein C; *GSK3*, glycogen synthase kinase-3; *CK1*, casein kinase 1; *TEADs*, transcriptional enhancer activator domains; *Runx2*, runt-related transcription factor 2; *Sp7/Osx*, transcription factor Sp7 or Osterix; *TCF1/LEF1*, specific T-cell factor/factor 1 transcriptional factor; *MMP13*, matrix metallopeptidase 13; *RANK-L*, receptor activator for nuclear factor κB ligand; *OCN*, osteocalcin; *OPN*, osteopontin; *ALP*, alkaline phosphatase; *OPG*, osteoprotegerin; *Col1*, type 1 collagen; *IGFBP-4*, insulin-like growth factor binding-protein 4.

Among the first human recombinant proteins selected for use in bone tissue engineering were BMPs, in particular BMP-2 [41]. Recombinant human BMP-2 (rhBMP-2) was approved by the Food and Drug Administration (FDA) and used clinically. The mechanism of action of the two most studied BMPs, BMP-2 and BMP-9, is shown in Figure 1.

Though rhBMP-2 shows a well-documented ability to induce bone formation, it has a very short half-life, requiring clinicians to use supraphysiological doses, and even its uncontrolled release in vivo could lead to ectopic bone formation, inflammation, and increased cancer risk [42].

This reinforces the idea that this recombinant protein must be applied through tissue engineering strategies, namely attached to matrices that slowly releases it in situ. The scaffolds act in these situations, not only as structures promoting tissue neoformation, but also as favoring the release of recombinant proteins at the site where they are implanted. A novel rhBMP-2-atelocollagen composite scaffold implanted in an experimental bone defect in rats induced intense new bone formation activity as demonstrated by the expression of bone formation markers, alkaline phosphatase (ALP), osteocalcin (OCN), osteopontin, and bone sialoprotein, detected by reverse transcription-polymerase chain reaction and immunohistochemistry [43]. In addition, other research groups proposed to attach rhBMP-2 to polimeric lactic scaffolds (PLLA), with encouraging preliminary results [44].

More recently, a polydopamine (pDA)-assisted BMP-2-derived peptide (named as P24) was developed by a surface modification strategy for attachment to a scaffold consisting of nano-hydroxyapatite (nHA)/recombinant human-like collagen (RHLC)/poly (lactic acid)(PLA), to improve its osteogenic capacity [45]. The immobilization efficiency and release kinetics of P24, and in vitro osteoinductive activity of the nHA/RHLC/PLA-pDA-P24 scaffold were examined in rat-derived mesenchymal stem cells (rMSCs) in vitro, showing increased ALP activity and *mRNA* expression of bone specific markers, compared with non-P24-loaded nHA/RHLC/PLA scaffold. Moreover, in vivo, nHA/RHLC/PLA-pDA-P24 scaffolds significantly enhanced bone regeneration in a rat critic-sized calvarial defect [45].

Furthermore, a pioneering study has recently compared the osteogenic potential of rhBMP-2 and rhBMP-9 [46]. In vitro, undifferentiated mouse ST2 stromal bone marrow cells were seeded onto bovine-derived natural bone mineral (NBM) particles used as scaffolds for the different BMPs. Neither rhBMP2 nor rhBMP9 influenced cell attachment to the scaffold particles, but both BMPs stimulated cell proliferation. All concentrations of both rhBMPs tested were able to significantly induce *mRNA* levels of Runx2, COL1a2 and OCN, but only rhBMP9 significantly upregulated ALP *mRNA* levels (up to eightfold), and ALP staining (up to 25-fold). Subsequent work by members of the same group with a fibrin sealant as a carrier system of rhBMP-9 demonstrated its high osteogenic potential [47].

A very recent study has investigated the bone formation capacity of rhBMP-2 absorbed in a collagen sponge (ACS), and proposed that these scaffolds could be a successful strategy in patients with osteonecrosis of the jaw [48]. Another recent strategy has exploited the ability of glycosaminoglycan hydrogel scaffolds (CS-GAG) to cell encapsulation, providing an extracellular matrix (ECM)-like microenvironment. In a very original experiment, MSCs cells were induced to overexpress rhBMP-2, and were then seeded in a CS-GAG hydrogel. The hydrogels were delivered in electrospun polycaprolactone nanofiber meshes, so that the release of rhBMP2 would occur locally as required at the bone defect. When applied to a model of critical bone injury, it was efficient to heal the latter, in terms of increased bone volume, strength, and stiffness of newly formed bone [49]. Moreover, synthetic PEG-based hydrogels, as biocompatible and tunable cell niches, were engineered to fast release platelet-derived growth factor (PDGF-BB) and sustained delivery of low-dose BMP-2. These hydrogels promoted bone formation, enhancing the effect of BMP-2 [50].

In summary, although BMP-2 and BMP-9 actively participate in processes related to bone formation and repair (Figure 1), due to physiological, cost, and adverse reaction reasons, their direct delivery at the sites of injury is inappropriate. Therefore, a variety of maneuvers have been developed, including synthesis of chimeric BMP-containing proteins or to promote their binding to scaffolds for their controlled release, to make them useful as bone tissue engineering strategies (Table 1).

## 6. Collagen-Based Recombinants

Currently, collagen used in tissue engineering applications are derived from animal tissues, which raises concerns related to the quality, purity, and predictability of their performance, as well as the risk of transmission of infectious agents and precipitating immune reactions. Therefore, in recent years, the interest of collagen in tissue engineering focused on the development of recombinant sources of human collagen that provide a predictable and chemically defined source of purified human collagen free of animal components [53]. The advantage found in this type of scaffold is that, in addition to being perfectly biocompatible, it has the ideal chemical and mechanical properties to replace the extracellular matrix of damaged tissue, such as structure, strength, mechanical integrity, and porosity, that allows cellular infiltration and nutrient diffusion required for osteoinduction and osteodifferentiation. Mechanical properties are a critical factor for cell adhesion, growth, and stem cell differentiation [54].

In 2004, Yang et al. [41] used recombinant human type I collagen (RCP) as a scaffold. Its production was achieved by cross-linking followed by lyophilization, forming a 3D porous structured scaffold with extensive surface areas, compared to those observed in type I collagen of animal origin, to allow cell proliferation, in vitro and in vivo. In vitro activity was analyzed in cell cultures for three months, considering osteogenic differentiation and mineralization. In the in vivo studies, using a cranial lesion in rats, the biocompatibility of the scaffold with mild and insignificant inflammation was demonstrated. Thereafter, Wang et al. [55] performed in vitro and in vivo studies, with an interesting scaffold produced with a human recombinant type 1 collagen nanohydroxyapatite-poly (lactic acid) composite. It was tested in vivo in rabbit radial defects that healed 24 weeks after surgery. These studies demonstrate that scaffolds with this recombinant collagen are similarly efficient as those containing collagen of animal origin, but avoid potential hazards (e.g., infection by viruses).

Pawelec et al. [54], in 2017, investigated the properties of recombinant type I collagen-based scaffolds by comparing three cross-linking procedures, using dehydrothermal, hexamethylene diisocyanate or genipin, to evaluate whether the chemical properties of the scaffolds could vary depending on the treatment. The mechanical properties were not significantly altered. The chemistry of the scaffolds was changed, affecting properties such as water uptake, and initial adhesion of human mesenchymal stem cells (MSCs); genipin crosslinking supported the lowest MSC adhesion. In vitro analysis using hMSC cultures showed that, regardless of the cross-linking method, the scaffolds can support osteoblast differentiation and mineralization.

In another study, three types of RCP scaffolds enriched in arginine-glycine-aspartate sequence were evaluated: one scaffold with non-mineralized RCP, another with mineralized RCP, and the third one with mineralized RCP in the presence of magnesium, all three with 3D isotropic porous structure and produced by lyophilization [35]. In vitro studies using MSC cultures revealed that the latter scaffold induced cell growth and increased gene and protein expression of osteogenic markers, best mimicking bone mineral composition, thus the most promising candidate for future clinical applications.

In 2017, Suarez-Muñoz et al. [56] produced a new microcarrier type constituted of a recombinant collagen I peptide This microcarrier allows the GMP-compliant upscaling of scaffold and cell production, as needed for cell delivery in a clinical scenario.

Pallabi et al. [57] developed collagen and elastin-like polypeptide (ELP)-based bone regenerative hydrogels loaded with recombinant human bone morphogenetic protein-2 (rhBMP-2) with mechanical properties suitable for osteogenesis.

In an interesting work in 2020, a recombinant protein based on collagen type I (RCPhC1) was functionalized with photo-cross-linkable methacrylamide (RCPhC1-MA), norbornene (RCPhC1-NB), or thiol (RCPhC1-SH) functionalities to enable high-resolution 3D printing via two-photon polymerization (2PP). 2PP is a lithography-based 3D printing method allowing the fabrication of 3D structures with sub-micrometer resolution. While only in vitro results have been shown so far, in which cells successfully grew and proliferated in these scaffolds, this development was a breakthrough in the use of recombinant matrices in 3D systems for use in tissue engineering [58].

In summary, collagen has an innate biocompatibility, making it attractive for bone tissue engineering efforts. Collagens serve as scaffolds for the attachment of cells and matrix proteins and the production of scaffolds based on recombinant collagen protein represents a simple, economical, and very useful possibility to be applied in bone regenerative medicine by means of tissue engineering strategies (Table 2).

## 7. Elastin-like Recombinamers (ELRs)

Certain polymers can be produced as recombinant chimeric proteins from a synthetic DNA construct, so called recombinamers [59]. Due to recent advances in molecular biology and biotechnology, the degree of complexity and control achieved in the final composition of recombinamers can be much higher than that achieved by the most advanced chemical methods in polymer science [60]. This allows the incorporation of any function present in natural proteins, such as cell adhesion sequences, specific protease targets, or higher molecular weight domains.

The family of ELRs have proven great application and relevance [59,60]. The composition of ELRs, based on the repetition of natural elastin sequences, L-Val-L-Pro-Gly-X-Gly, where X can be any amino acid except L-Pro, endows the material with a series of properties that are not easy to find in other polymer families: extreme biocompatibility, strong responsiveness to external stimuli, self-assembly, and adequate mechanical properties of its hydrogels. ELRs provide transient mechanical support and act as a vehicle that introduces a number of specific tissue repair bioactivities. The combination of these properties allows these materials to mimic the rich and complex functionality of the extracellular matrix [60,61,62,63,64,65]. With a different approach, amphiphilic systems based on ELR copolymers of order four or higher gave rise, through self-assembly processes, to the formation of physical gels with excellent mechanical properties. These gels have a marked elastic nature, and the gelation temperature can be adjusted to the desired value by controlling the polymer composition. In addition, the gelation time of this type of system is very short, which is very important to avoid dilution of the implant in the biological environment, which would diminish its effectiveness [66].

An alternative way to obtain non-cytotoxic and biocompatible hydrogels with application in tissue regeneration from ELRs exploits an innovative strategy in the generation of hydrogels. The biofunctionality of this type of hydrogels lies in the introduction of bioactive sequences in the composition of the biopolymers [67]. Recently, two bioactive ELRs were developed, one including the osteogenic and osteoinductive BMP-2, and the other the Arg-Gly-Asp (RGD) cell adhesion motif. These two ELRs were mixed, obtaining a hydrogel scaffold that was considered to act as an extracellular matrix carrying BMP-2, feasible for bone tissue engineering strategies. This scaffold exhibited excellent cytocompatibility, and the culture of cells on RGD-containing ELRs resulted in optimal cell adhesion. When implanted in a rabbit bone injury model, complete regeneration of the defect—confirmed by radiography, computed tomography, and histology—was demonstrated [51] (Table 1).

In recent years, new biomaterials based on ELRs that are genetically engineered using recombinant technology, bioproduced by fermentation in *E.coli*, have been developed with proven qualities in regenerative medicine [68]. One of the most important characteristics of these new ELR polymers is that they can be specifically designed and tailored for each application. In the first place, these materials have demonstrated their bioactivity and efficacy with general adhesion sequences (RGD) [69], and with specific adhesion sequences such as REDV [70]. Their excellent biocompatibility [71] and their ability to induce angiogenesis [72] have also been demonstrated. In addition, when used as implants in a defect, these biomaterials are able to recruit cells from the surrounding tissues and induce tissue regeneration in the area of the lesion, without the need to introduce cells with the implant [73,74]. It is also noteworthy how its degradation rate can be regulated by specific sequences mediated by elastases [75].

One of the key properties that regenerated tissue might achieve is that newly produced cells would be able to produce an extracellular matrix containing elastin/collagen similar to that of native tissue. Generally, cells embedded in various biomaterials generate collagen but not elastin, and therefore the mechanical properties are not adequate for the final application [76]. There are studies showing how embedded cells in ELRs are able to generate both collagen and elastin distinguishable from that of ELR [77].

The technology required for the creation of hydrogels from ELRs is available in the literature. Chemical modification of these polypeptides is performed by introducing reactive groups to form chemical hydrogels through a “click chemistry” reaction and, more specifically, through a Huisgen cycloaddition under fully cytocompatible conditions and without the presence of any initiator or catalyst [67]. Hydrogels formed through this reaction have shown mechanical properties similar to those found in natural tissues, and a high biocompatibility, as mentioned above [67].

More specifically, in relation to skeletal regeneration, nanoparticles have been developed from ELR capable of effectively encapsulating BMPs and controlling their release for 14 days. Moreover, the osteoinductive activity of these growth factors was evidenced by the induction of ALP activity and osteogenic mineralization in C2C12 cells [78]. Hydrogels have displayed also osteoinductive effects in several studies, due to their ability to recruit osteoprogenitor cells from native tissues surrounding the lesion [51]. Finally, different hydrogels have been prepared from ELR with embedded cells, and have recently been used in different studies on the regeneration of osteochondral defects in rabbits, with very promising results [79,80].

## 8. Recombinant Peptides Targeting Integrins

Integrins are a family of heterodimeric transmembrane glycoproteins that are known to mediate cell–cell and cell–matrix interactions. The path of integrin research applied to bone tissue regeneration has just begun and is very promising, because the interaction of α2β1 integrin with collagen I is a crucial signal for osteoblastic differentiation and mineralization. Integrins may mediate the differing interactions of cells of the osteoblast and osteoclast lineages with the matrix of bone [81]. Shekaran et al. [52] developed a protease-degradable poly(ethylene glycol) (PEG) synthetic hydrogel, functionalized with a triple helical α2β1 integrin-six aminoacid-peptide (GFOGER) as a BMP-2 delivery scaffold. First, the research engineered and produced a synthetic collagen I-mimetic-containing peptide, GGYGGGP(GPP)_5_GFOGER(GPP)_5_GPC, which recapitulates the triple helical structure of native collagen and binds α2β1 integrin with high affinity and specificity. Then, rhBMP2 was linked to this construct using GCRDVPMSMRGGDRCG (VPM) as a cross-linker peptide. In a murine non-healing radial bone defect, this composite hydrogel promoted osteoprogenitor cell recruitment to the defect site and produced robust repair and bone bridging. Thus, this type of hydrogel displayed intrinsic osteogenic activity, underwent rapid degradation in vivo, and bridged a critical-sized bone defect at low BMP-2 doses, which is of the utmost importance.

More advanced studies on recombinant integrins were performed, such as computational simulations for silk–silica–integrin binding, which showed activation of αVβ3 integrin in contact with silica. This integrated experimental and computational research provides novel insights into interactions that regulate osteogenesis towards more efficient biomaterial designs [82]. Recently, these ideas were reinforced by stating that integrin-functionalized hydrogels might modulate the in vitro behavior of hMSCs. Thus, it was demonstrated that integrin-specific hydrogels regulate hMSC adhesion, paracrine signaling, and osteoblastic differentiation in vitro. Furthermore, hydrogels presenting previously named six aminoacid peptide GFOGER prolong hMSC survival and engraftment in a segmental bone defect, resulting in improved bone repair [83]. In a recent work, Xin et al. [84] compared the bioactivity of a cyclic peptide, cRRETAWA, which targets α5β1integrins, with those of the widely used RGDS motif that binds to many different integrins in hMSC cultures, and found its high efficiency in inducing hMSC osteogenic differentiation.

The ligands which integrins recognize are conserved amino acid sequences found in many components within the extracellular matrix. The clustering of integrins has a large impact on the downstream cellular effects, increasing signalling effectiveness, in times independent of the global density of ligand present [85]. The use of recombinant integrins as the basis for new scaffolds is intended to address cellular processes to improve bone tissue regeneration. However, since the in vivo concentrations of ligands that enable cluster-enhanced integrin signaling are variable, much research remains to be done to apply this concept to scaffolds applied to bone tissue regeneration in pre-clinical models [86].

## 9. Recombinant Spider Silk

Spider silk is made of protein fibers spun by spiders. Spiders use their silk to make webs or other structures, which function as sticky nets to catch other animals, or as nests or cocoons to protect their offspring or to wrap up prey. The properties of native spider silk vary within and across species due to the presence of different genes containing conserved repetitive core domains encoding a variety of silk proteins. Spider silk is biocompatible, strong and elastic, and hence an attractive biomaterial [87,88]. The most used recombinant spider silk proteins are based on sequences from Nephila clavipes or Araneus diadematus [89]. These proteins can form various types of scaffolds—fibers, films, 3D-foams, hydrogels, tubes, and microcapsules—because they can self-assemble.

Two independent groups have made great progress in bone tissue engineering based on the structure of these silk proteins. Bini et al. [90] generated two recombinant proteins from the consensus sequence of the major component of dragline silk from Nephila clavipes, including RGD cell-binding domains. The proteins produced were processed into films and fibers. It was observed that these scaffolds increased the differentiation of human marrow stromal cells into osteoblastic progeny in the presence of osteogenic stimulants. In addition, Yang et al. [53] generated biomimetic calcium phosphate coatings on recombinant spider silk fiber, showing that they supported the attachment and growth of hMSCs.

In 2011, an interesting recombinant chimeric protein of spider silk (the consensus repeat for the Nephila clavipes spider dragline protein) and bone sialoprotein (BSP) was designed. The clone carrying the DNA sequence coding for BSP was inserted in the vector pET30L (Novagen, San Diego, CA, USA) carrying the silk block copolymer. After the expression and purification of the chimeric protein, a film chimeric scaffold was obtained that had more elastic modulus than the film of spider silk alone without the BSP. This protein retained mineralization potential and sustained human mesenchymal stem cell proliferation and differentiation into the osteogenic lineage [91].

An interesting contribution was made more recently, using a scaffold produced from a recombinant spider silk, based on the consensus motif of the repetitive core domain of one of the major ampullate silk fibroins of the garden cross spider, *A. diadematus* fibroin 4 [92]. This recombinant protein contains sixteen repeats of the polypeptide module C (GSSAAAAAAAASGPGGYGPENQGPSGPGGYGPGGP amino acid sequence), referred to hereafter as eADF4(C16). It was found to display multiple carboxylic acid moieties capable of binding calcium ions, which would favor the mineralization process. In vitro studies with MSCs seeded on these scaffolds showed a significant increase in ALP activity, as well as its potential in bone tissue engineering.

Moreover, a scaffold was produced from cloning a chimera protein containing the spider silk inspired domain (SGRGGLGGQG AGAAAAAGGA GQGGYGGLGSQGT)_15_—serving as an organic scaffold to control material stability and to allow multiple modes of processing—and the HA binding domain VTKHLNQISQSY (VTK), providing control of osteogenesis [93]. These scaffolds increased differentiation of bone marrow-derived hMSCs into osteoblastic progeny.

Recently, the spider silk proteome has provided the structural characterization of the entire structure of *Nephila clavipes* flagelliform spidroin [94]. The long sequence of this flagelliform silk protein presents 45 hydroxylated proline residues, each located in the GPGGX motif, which may contribute to explain the mechanoelastic property of these fibers. This is a very interesting property to be present in scaffolds to be applied in bone tissue engineering. Knowing this sequence could be applied to the construction of recombinant chimeric proteins with this property.

A recent study by Neubauer et al. [95] has reported the development of a new chimeric protein adding coding sequences of bone-related proteins, namely osteopontin and sialoprotein, to spider silk proteins.

The properties of native spider silk vary within and across species due to the presence of different genes encoding a variety of silk proteins with conserved repetitive core domains [96]. The latter can be used as building blocks for new silk-based biomaterials with varying mechanical properties. This knowledge opens the door to its putative use with other osteogenic proteins, like BMP-2, in scaffolds for application in bone tissue engineering.

## 10. Future Perspectives

The development of new scaffolds using recent advances in molecular biology undoubtedly provides novel strategies in tissue engineering [97]. In particular, scaffolds with recombinant proteins for bone tissue engineering application have broad prospects and wide frontiers to explore. Scaffolds based on human recombinant R-spondin (RSPO) proteins are a good example in this respect. RSPO comprises a family of secreted proteins with important roles in cell proliferation and differentiation [98]. RSPOs display characteristic domains which are conserved among vertebrates, such as: (1) a thrombospondin 1 repeat domain; (2) a cysteine-rich furin-like domain; (3) a basic amino acid-rich domain of variable length in the C-terminal region; and (4) a hydrophilic signal peptide sequence [99]. They are broadly expressed during skeletal development, and modulate osteoblastogenesis and bone formation by inducing the canonical WNT/β-catenin pathway [100]. A stable platform for the production of recombinant hRSPO1 from HEK293 cells was generated, leading to the production of a purified, fully characterized bioactive protein product for bone tissue engineering applications [101].

From the perspective of recombinant protein strategies, new technologies have emerged that are still in their infancy but have a high potential in bone regenerative medicine. Particularly noteworthy in this regard, a very recent report from Hsu et al. [102] has exploited the fact that BMP2 is known to induce the expression of its antagonist noggin that self-restricts its bioactivity. Therefore, they proposed that a CRISPRi-based system providing noggin inhibition concurrently to BMP2 overexpression might improve bone healing. In fact, this complex system was found to stimulate osteogenic differentiation of adipose-derived stem cells in vitro, and promoted the mineralization and repair of a calcaneal bone lesion in vivo.

Indeed, bone tissue engineering using molecular biology strategies and recombinant protein production is advancing rapidly. The advent of recombinant DNA strategies as well as the development of genomic strategies that allow the design of oligomeric sequences coding for proteins of interest represent great improvements for recombinant protein production. In addition, production of chimeric proteins would provide alternatives for the development of multifunctional scaffolds to promote osteogenesis at the site of bone injury. This is particularly important to repair non-union fractures and to avoid double surgeries required by autologous bone replacement [103,104,105].

Novel protocols applying tissue engineering strategies based on recombinant proteins for bone tissue repair in the clinical practice are in constant development, with very encouraging results [1,106,107,108,109]. Current strategies in this regard intend to promote a close relationship between basic researchers who develop recombinant scaffolds and physicians who face the different types of problems associated with bone pathology [110,111,112]. In this way, knowledge of the metabolic pathways supporting bone health, such as those shown in Figure 1, facilitates the development of recombinant scaffolds to meet specific needs, without undesirable side effects, for each type of bone lesion.

All of the above indicates that bone tissue repair based on recombinant molecular biology strategies has a promising future in bone regenerative medicine. However, use of recombinant chimeric proteins for use in medical practice requires further exploration of the physiological mechanisms of bone repair in order to generate bone tissue repair biomolecules that possess the appropriate structure to be delivered at the site of injury. By this regard, researchers have been increasingly focusing on recombinant materials that can be produced with full reproducibility, and can be designed according to specific needs to form scaffolds. The present updated data provide a synthetic view of the most relevant advances in this respect, focusing on those already achieved or those that are currently in progress in bone tissue regeneration.

## Figures and Tables

**Table 1:** Outstanding work applying recombinant bone morphogenetic proteins (BMPs) in tissue engineering strategies.

Type of Scaffold	In Vitro	In Vivo	Results	References
Collagen and BMP2	hMSC	Cranial defects in rats	Satisfactory activity of alkaline phosphatase. Histopathological study and nuclear magnetic resonance imaging repair of the upper bone defect with the association of BMP-2. Insignificant inflammation.	[41]
Mineralized recombinant human-like collagen, nano-hydroxyapatite/recombinant human-like collagen/poly (lactic acid) nHA/RHLC/PLA scaffold with polydopamine (pDA)-assisted BMP-2-derived peptide (named as P24) as surface modification strategy	Rat MSC	Cranial defects in rats	Increased ALP activitiy and mRNA expression of osteo-specific markers of the nHA/RH)LC/PLA-P24 and non-P24-loaded nHA/RHLC/PLA groups. In vivo, it is demonstrated that the nHA/RHLC/PLA-pDA-P24 scaffolds significantly enhanced bone regeneration of rat cranial defects.	[45]
Atellocollagen and BMP-2	No	Rats	Expressions of bone phenotypic markers, alkaline phosphatase, osteocalcin, osteopontin, and bone sialoprotein were detected by reverse transcription-polymerase chain reaction and immunohistochemistry. Mineralization and the expressions of key bone proteins were demonstrated in chondroblasts and osteoblasts at 7 to 14 days of culture.	[43]
NBM and BMP-2	Mouse ST2 stromal bone marrow cells seeded on natural bone mineral of bovine origin	No	All concentrations of rhBMPs were able to significantly induce mRNA levels of Runx2, COL1a2 and OCN, but only rhBMP9 was able to significantly upregulate mRNA levels of ALP up to eight-fold, and ALP staining up to 25-fold, when compared to rhBMP2.	[47]
Two bioactive ELRs were developed, one including the osteogenic and osteoinductive bone morphogenetic protein-2 (BMP-2) and the other the Arg-Gly-Asp (RGD) cell adhesion motif. These two ELRs were mixed, obtaining a hydrogel scaffold	Bone marrow human MSC	Rabbit lateral distal metaphysic of the femur	In vitro, excellent cytocompatibility observed, and the culture of cells on RGD-containing ELRs resulted in optimal cell adhesion; in vivo, complete regeneration of the defect confirmed by radiography, computed tomography, and histology was demonstrated.	[51]
Protease-degradable poly(ethylene glycol) (PEG) synthetic hydrogel functionalized with a triple helical, α2β1 integrin-specific peptide (GFOGER) as a BMP-2 delivery scaffold	hMSC	Murine radial bone defect	These hydrogels promoted osteoprogenitor cell recruitment to the defect site and produced robust repair in a murine non-healing radial bone defect. These hydrogels displayed intrinsic osteogenic activity.	[52]
Glycosaminoglycan scaffolds (CS-GAG) hydrogelMSCs cells were modified to overexpress BMP-2 which are then seeded in a CS-GAG hydrogel. ectrospun polycaprolactone nanofiber meshes	Human umbilicas (uMSC) and bone marrow (bmMSC)	Nude rats; critically -sized defects in the mid-diaphysis of the femur	Extended release of rhBMP-2 from CS-GAG scaffolds and further extended release from CS-GAG gels seeded with BMP-2 MSC was demonstrated. In vivo, in bone injury, very good results were obtained, as measured by bone volume, strength, and stiffness.	[49]

**Table 2:** Outstanding work applying recombinant human collagen in tissue engineering strategies.

Scaffolds	In Vitro	In Vivo	Results	References
Recombinant human type I collagen achieved by cross-linking followed by lyophilization, forming a 3-D porous structured scaffold	Yes	Cranial defects in rats	Osteogenic differentiation of stem cells. Mineralization increased with the scaffolds. High biocompatibility in vivo.	[41]
Recombinant human type I collagen–nanohydorxyapatite-poly(lactic acid) composite	Yes	Radial defects in rabbits	Osteogenic differentiation. Similar effects to collagen of animal origin, but without potential hazards.	[55]
Recombinant type I collagen-based scaffolds, obtained by three cross-linking procedures, using dehydrothermal, hesamethylene diisocyanate or genipin	Yes	No	Genipin crosslinking recombinant type I collagen scaffolds supported the lowest MSC adhesion. All the cross-linking methods produced scaffolds that support osteoblast differentiation and mineralization.	[35]
Recombinant collagen and elastin-like polypeptide (ELP)-based bone regenerative hydrogels loaded with recombinant human bone morphogenetic protein-2 (rhBMP-2)	Yes	No	Collagen-ELP hydrogels had a significantly higher modulus of 35 ± 5 kPa compared to collagen-only hydrogels. In vitro osteogenic markers, alkaline phosphatase and osteocalcin, were expressed.	[57]
Recombinant collagen type I was functionalized with photo-cross-linkable methacrylamide (RCPhC1-MA), norbornene (RCPhC1-NB), or thiol (RCPhC1-SH) functionalities to enable high-resolution 3D printing via two-photon polymerization (2PP)	Yes	No	Hydrogels developed were processable via 2PP and proved to be a perfect alternative to serve tissue engineering applications.	[58]

## Data Availability

All the data mentioned in this review are published in different high impact international journals of common access for researchers and readers of scientific journals, and their access is indicated in the bibliography. The produced image presented was originally produced by the authors.
